# Can Digit Ratio (2D:4D) Be Indicative of Predispositions to Autoimmune Thyroid Diseases in Women - Hashimoto Thyroiditis and Graves’ Disease?

**DOI:** 10.3389/fendo.2022.914471

**Published:** 2022-06-30

**Authors:** Barbara Święchowicz, Anna Kasielska-Trojan, John T. Manning, Bogusław Antoszewski

**Affiliations:** ^1^Plastic, Reconstructive and Aesthetic Surgery Clinic, Institute of Surgery, Medical University of Lodz, Lodz, Poland; ^2^Applied Sports, Technology, Exercise, and Medicine (A-STEM), Swansea University, Swansea, United Kingdom

**Keywords:** digit ratio, Hashimoto thyroiditis, Graves’ disease, autoimmune thyroid diseases, estrogen

## Abstract

Hashimoto thyroiditis and Graves’ disease are autoimmune thyroid diseases which occur much more frequently in women than in men. Estrogen receptors are found in the thyroid gland and can modulate the gland’s function. Digit ratio (2D:4D) is thought to be a negative correlate of prenatal testosterone and a positive correlate of prenatal estrogen. This study aimed to examine a relationship between right and left 2D:4D in women with Hashimoto thyroiditis and Graves’ disease. The cross-sectional study included 106 women with autoimmune thyroid disease: 73 women diagnosed with Hashimoto thyroiditis and 33 women with Graves’ disease, together with 70 healthy women as controls. Second and fourth digit length, weight, height were measured directly, and 2D:4D and BMI were calculated. Compared to controls, right and left 2D:4D were significantly higher in women with Hashimoto thyroiditis and lower in women with Graves’ disease, the effects were higher for right 2D:4D. The mean length of right 4D was significantly lower in the examined women with Hashimoto thyroiditis than in Graves’ disease. Higher right and left 2D:4D in women with Hashimoto thyroiditis suggests that prenatal exposure to high levels of estrogens relative to testosterone may play a role in the development of this disease. Lower right and left 2D:4D in women with Graves’ disease suggest a role of high prenatal androgens relative to estrogens in Graves’ disease pathogenesis.

## 1 Introduction

Autoimmune thyroid disease (AITD) is a category of disorders in which the immune system targets thyroid cells (1). The most common AITDs are Hashimoto thyroiditis and Graves’ disease. Both have a higher prevalence in women than men. However, in Hashimoto thyroiditis, women’s dominance is the highest as it is 10-15 times more frequent in women than in men (2, 3).

Hashimoto thyroiditis affects mainly middle-aged women, usually at the age between 30-50 years but can also be present in children (2). This age range indicates that the disease could be sex-hormone dependent. The thyroid gland dysfunction can be symptomatic (0.1-2% of the population) or subclinical (10-15%) (4). Hashimoto thyroiditis involves elevation of thyroid peroxidase antibodies (TPOAb) and thyroglobulin antibodies (TgAb) and usually is clinically manifested as hypothyroidism (5). In its autoimmune pathophysiology, the disease is T-helper 1 (Th1) cell-mediated, however the trigger of the disease seems to depend on hormonal factors – i.e. estrogen level changes (estrogen dominance) (6). Helper T cells (Th) have the expression of CD4 and can be divided into Th1 and Th2. Th1 lymphocytes produce proinflammatory cytokines, killing intracellular parasites and taking part in autoimmune responses. The main cytokine of Th1 is interferon-gamma (7).

Pathophysiologic indicators in Graves’ disease include anti-thyroid stimulating hormone receptor antibodies (TRAb). Thyroid peroxidase antibody (TPOAb) and thyroglobulin antibody (TgAb), characteristic of Hashimoto disease, can also be present. The clinical manifestation of this disease is related to hyperthyroidism: goiter, anxiety, weight loss, tachycardia, dermopathy and ophthalmopathy. Hyperthyroidism is the most common cause of toxic goiter which occurs approximately in 1 to 200 people The clinical manifestation depends on the age of disease onset. Palpable goiter occurs more frequently in patients younger than 60 years old. Toxic goiter affects 1 to 200 people in the United States (8). Weight gain can be observed in 10% of cases. Thyroid dermopathy is very rare and occurs in 2 to 3% of cases (9). Graves’ orbitopathy has the incidence rate of 16 women and 3 men per 100,000 (10) and can occur in 40% of patients with Graves’ disease (11). Symptoms like acropachy, osteopathy and onycholysis (Plummer nails) are very rare (9). Graves’ disease occurs eight times more often in women than in men. The peak incidence of Graves’ disease is at the age range of 30-60 years (12). A key role in the autoimmune pathophysiology is attributed to the systemic shift of cytokine production toward the Th2 response, which is promoted by androgens (13).

The fact that Hashimoto thyroiditis and Graves’ disease are sex-hormones dependent is indicated by observations that the immune system is sexually dimorphic in its effects. In general, estrogen has an immunoenhancing effect on the immune system (within its normal “non-pregnancy” ranges), while androgen is immunosuppressive (14). There are many theories concerning pathophysiologic mechanisms by which sex steroids influence the immune system. One such explanation involves the expression of estrogen receptors on most of the immune cells (15, 16). Estrogen suppresses B cell lymphopoiesis and differentiation from pro-B to pre-B cell stage and allows the escape of autoreactive B-cells (17). What is more, it increases Th1 differentiation (but in bone and the central nervous system, the effect is opposite). In contrast, androgens inhibit Th1 and promote Th2 (17).

The digit ratio (2D:4D) is the relative lengths of the second (2D) and fourth (4D) digits. Previous studies showed the sexual dimorphism of the ratio and presented evidence for its relationship with prenatal sex steroids exposure (18–20). Mean 2D:4D is higher in females than males. In fetal life, low estrogen level and high testosterone relate to low digit ratio, and high estrogens and low testosterone level are thought to correlate with a high digit ratio. In addition, right 2D:4D is often found to be lower than left 2D:4D suggesting the former is more sensitive to prenatal sex steroids (18). Thus, high Δ r-l 2D:4D (right – left 2D:4D) is also thought to indicate high prenatal estrogen and low prenatal testosterone (19, 21). Studies analyzing digit development in an animal model in mice confirmed this hypothesis and showed that 2D:4D depends on the androgen and estrogen receptors situated mainly in the ring finger and also in the index finger (22). Currently, there is considerable evidence for the links between 2D:4D and prenatal sex steroids in humans (23) (but see (24) for an alternative view).

With regard to links between 2D:4D and thyroid disease, Manning (25) (pp. 62-63) has suggested that 2D:4D may be correlated with thyroid function. The principal products of the thyroid gland are the hormones thyroxine (T4) and the more active triodothyronine (T3) and production is dependent on thyroid stimulating hormone (TSH) which is secreted by the pituitary gland. The fetal thyroid gland reaches maturity by week 11–12, and begins to secrete thyroid hormones by week 16. During early pregnancy T4 finds its way to the fetus from maternal serum (26). Estrogen and testosterone exert their influence on thyroid function by altering levels of thyroxine-binding globulin (TBG). Estrogen administration causes an increase in serum TBG concentration, androgen administration results in a decrease in this binding protein (27). Manning pointed out the importance of sex steroids in the regulation of the thyroid and cited data from Chinese children showing a positive correlation between right 2D:4D and TSH (25)]. If such a link is confirmed it may indicate that in children with high 2D:4D elevated levels of TSH production from the pituitary gland are necessary to maintain normal T4 production (25).

Considering diseases of the thyroid, despite the estrogen/testosterone-induced modulation of TBG concentrations, subjects with normal thyroid glands maintain euthyroidism without changes in their serum free T4 or TSH levels. In contrast, the administration of sex steroids to patients with thyroid diseases causes significant biochemical and clinical alterations. For example, estrogen administration to hypothyroid women causes a decrease in serum-free thyroxine levels and a simultaneous increase in serum TSH levels (27).

There are no studies on associations between AITD and biomarkers of prenatal sex steroids. Tabachnik et al. showed that women with different thyroid disorders had a higher digit ratio, however they did not interpret this observation (28). It is well known that postnatal estrogens have an impact on the thyroid gland and have the potential to promote benign and malignant thyroid cell growth *via* membrane-bound estrogen receptors (29). Moreover, estrogen can modulate the immune response by enhancing humoral responses (30). Nothing is known about the possible impact of prenatal sex steroids exposure on the risk of AITD. Therefore, we conducted this study to investigate 2D:4D in the AITD – Hashimoto thyroiditis and Graves’ disease. We hypothesized that high estrogen and low testosterone exposure during prenatal life may increase the risk of AITD in later life. This study aimed to examine right and left 2D:4D in women with Hashimoto thyroiditis and Graves’ disease in comparison to healthy controls.

## 2 Materials and Methods

### 2.1 Study Population

The study involved 106 women with AITD - 73 with a diagnosis of Hashimoto thyroiditis of the average age of 43.3 years (SD = 19.6) and 33 with a diagnosis of Graves’ disease of the average age of 54.8 (SD=12.9). Also 70 healthy women of the average age of 40.8 years (SD = 12.5) were included. All patients with thyroid disease were diagnosed by an endocrinologist and were included in this study only when a diagnosis of Hashimoto thyroiditis or Graves’ disease was confirmed. In addition, a detailed medical interview had also been conducted, and a clinical questionnaire included data concerning: age at diagnosis, family history of thyroid diseases, number of pregnancies, miscarriages, age at menarche, and other diseases. Women with other diagnosed endocrine disorders and diseases like polycystic ovary syndrome (PCOS) were excluded from the study, but patients with acne (without the diagnosis of PCOS) were not disqualified. Control women were recruited among age-matched Plastic Surgery Outpatient Clinic after excluding chronic diseases in this group. Those who reported hand/digit injuries were excluded from the study. It is known that 2D:4D varies across ethnic groups (18). All participants in the study were Caucasian.

The study was conducted according to the guidelines of the Declaration of Helsinki, and approved by the Ethics Committee of Medical University of Lodz (RNN/04/19KE, 15.01.2019). Written informed consent has been obtained from the patients.

### 2.2 Measurements

In the examined women and controls the following measurements were taken: body height (B-v), II and IV digits’ lengths (right (R) and left hand (L)) (2D R, 2D L, 4D R, 4D L) and body weight. On the basis of the measurements the following indices were calculated: BMI (weight [kg]/(B-v)^2^ [m^2^]), 2D:4D for the right (R) and left (L) hand (digit II length [cm]/digit IV [cm]) (2D:4D R, 2D:4D L) and Δ r-l 2D:4D (right 2D:4D minus left 2D:4D). All measurements were made directly with GPM anthropological instruments (sliding caliper, anthropometer, measuring tape). Finger lengths were also measured directly on the palmar side of the hand using anthropometric points lying on the digit axis: pseudophalangion (pph)—a point in the proximal finger crease, and dactylion (da)—the most distal point on the finger tip.

### 2.3 Statistical Analysis

Analysis was conducted with regard to differences in the studied anthropometric features (measurements and indices) between women with Hashimoto thyroiditis, women with Graves’ disease and control women. In order to compare the three groups by age, weight, height, BMI, and 2D:4D, we used a one-way univariate ANOVA or its nonparametric equivalent - Kruskal-Wallis test if the ANOVA assumptions were not met (normal distribution and homogeneity of variance).

Prior to a statistical analysis of the metric data, the normality of the distribution of the tested variables was examined by Kolmogorov-Smirnov test and the homogeneity of variance was assessed with the Brown-Forsythe test due to the fact that the analysed groups are not equal in size. In cases where significant differences were found, we used post-hoc tests: multiple comparisons of mean ranks and Tukey’s tests (HSD) for unequal groups. Discriminant analysis was performed to verify the influence of BMI on the observed correlations. Pearson’s or Spearman’s correlation coefficients were used to check associations between digits’ lengths and ratios and the age of the disease onset, the number of pregnancies, the age of menarche, and BMI in women with Hashimoto thyroiditis and Graves’ disease. Correlations between digits’ lengths and ratios and the history of miscarriages and concomitant diseases were analysed with t-test or Mann-Whitney test. The effect size for inter-group differences was evaluated with Cohen’s *d* for t-tests and Glass *r* for Mann-Whitney tests.

### 2.4 Results

#### 2.4.1 Age, Height, Weight and BMI in Women With Hashimoto Thyroiditis, Graves’ Disease and Control Women

The groups differed significantly in terms of age, weight and BMI. Women with Graves’ disease were older than women with Hashimoto thyroiditis and control women (mean age for Graves’ disease=57.76, Hashimoto thyroiditis=43.31, control=40.79) (p<0.01). Weight and BMI were higher in women with AITD than in control women (the mean BMI for the controls - 22.42 (SD 2.11), for women with Hashimoto thyroiditis - 26.12 (5.05) and for patients with Graves’ disease - 25.39 (4.41) (**Table 1**).

**Table 1:** Comparison of age, weight, height, BMI and digit measurements between women with Hashimoto thyroiditis, Graves’ disease and control women.

	H	df	p
age	16.50	2	0.0003
Hashimoto thyroiditis vs. Graves’ disease			0.0019
Hashimoto thyroiditis vs. control			1.0000
Graves’ disease vs. control			0.0003
weight	31.21	2	<0.0001
Hashimoto thyroiditis vs. Graves’ disease			1.0000
Hashimoto thyroiditis vs. control			<0.0001
Graves’ disease vs. control			0.0004
height	1.31	2	0.5198
BMI	27.17	2	<0.0001
Hashimoto thyroiditis vs. Graves’ disease			1.0000
Hashimoto thyroiditis vs. control			<0.0001
Graves’ disease vs. control			0.0027
2DR	2.26	2	0.3230
4DR	6.70	2	0.0350
Hashimoto thyroiditis vs. Graves’ disease			0.0423
Hashimoto thyroiditis vs. control			0.2682
Graves’ disease vs. control			0.8230
2DL	0.56	2	0.7542
4DL	3.67	2	0.1593

H - the Kruskal-Wallis H test, 2D – second digit, 4D – fourth digit, R – right, L - left.

#### 2.4.2 Digit Lengths and Ratios (2D:4D) in Women With Hashimoto Thyroiditis, Graves’ Disease and Control Women

Both right and left 2D:4D were different in all groups: the highest ratio was observed in women with Hashimoto thyroiditis and the lowest in women with Graves’ disease. Effect size for the right 2D:4D was higher than for the left (right ω^2 ^= 53.60%, left ω^2 ^= 37.94%) (**Tables 2**, **3**). The observed correlations were independent of BMI (Wilks’ lambda: for BMI – 0.84, p<0.0001, for 2D:4D R – 0.46, p<0.0001, for 2D:4D L – 0.61, p<0.0001). Moreover, we found that the mean length of 4D was lower in the examined women with Hashimoto thyroiditis than in the Graves’ disease group (*p*=0.04) (**Table 1**).

**Table 2:** Digit measurements and ratios in women with Hashimoto thyroiditis and Graves’ disease and control group.

	Hashimoto thyroiditis		Graves’ disease		Controls	
	mean	SD	mean	SD	mean	SD
2DR	64.96	9.94	64.70	8.41	68.11	3.82
4DR	62.13	9.95	67.15	8.87	66.86	3.69
2D:4D R	1.05	0.03	0.96	0.02	1.02	0.03
2DL	64.68	9.98	64.67	8.75	67.64	3.62
4DL	62.19	10.22	66.45	8.71	66.71	3.38
2D:4D L	1.04	0.04	0.97	0.04	1.01	0.03
Δ2D:4D	0.00	0.04	-0.01	0.03	0.01	0.03

2D – second digit, 4D – fourth digit, R – right, L – left, Δ2D:4D - right 2D:4D minus left 2D:4D.

**Table 3:** Comparison of digit ratios and delta between women with Hashimoto thyroiditis, Graves’ disease and control women (ANOVA).

	F	p	effect size ω^2^
2D:4D R	102.67	<0.0001	53.60%
Hashimoto thyroiditis vs. Graves’ disease		<0.0001	
Hashimoto thyroiditis vs. control		<0.0001	
Graves’ disease vs. control		<0.0001	
2D:4D L	54.81	<0.0001	37.94%
Hashimoto thyroiditis vs. Graves’ disease		<0.0001	
Hashimoto thyroiditis vs. control		<0.0001	
Graves’ disease vs. control		<0.0001	
Δ2D:4D	2.16	0.1179	1.31%

F - one-way ANOVA, 2D – second digit, 4D – fourth digit, R – right, L – left, Δ2D:4D - right 2D:4D minus left 2D:4D.

#### 2.4.3 Clinical Variables in Women With Hashimoto Thyroiditis, Graves’ Disease and Control Women

The age of disease onset, the age of the first menstruation and the number of pregnancies in the Hashimoto thyroiditis and Graves’ disease groups were similar (**Table 4**). There were no correlations between ratios and Δ r-l 2D:4D and the age of disease onset, number of pregnancies, age of menarche and BMI in any of the analysed groups. Positive family history for thyroid diseases was reported by 26 women with Hashimoto thyroiditis (mothers of 9 had AITD) and by 15 women with Graves’ disease (mothers of 7 had AITD).

**Table 4:** Age of the disease onset, menarche and number of pregnancies in women with Hashimoto thyroiditis and Graves’ disease.

		t	df	p	Cohen’s d
age of disease onset		-0.83	95	0.4091	0.18
		**Z**		**p**	**Glass r**
age of menarche number of pregnancies	* *	-0.55 -1.21	- -	0.5823 0.2248	0.10 0.15

*Mann-Whitney test, without *: t-test.

## 3 Discussion

There are many papers reporting on the association between sex-dependent diseases and digit ratio. They add to the knowledge about the possible pathophysiology of the diseases and present some clinical implications, e.g., they may help guide pharmacotherapy or its timing or be useful in determining specific groups of patients in whom therapies could be implemented earlier. However, to our knowledge, there have been no reports concerning the 2D:4D in AIT diseases, including Hashimoto thyroiditis and Graves’ disease. In this study, we found that women with Hashimoto thyroiditis have a higher right and left 2D:4D ratio than healthy women from the control group. It can suggest the thesis that high prenatal estrogen to testosterone exposure (e.g., due to estrogen receptors increased sensitivity or hyperestrogenic environment) can influence vulnerability to Hashimoto thyroiditis development in adult life. Moreover, women with Graves’ disease have lower 2D:4D (both right and left) than women in the control group and those with Hashimoto thyroiditis. This may be due to lower exposure to estrogen (or higher to androgens) during prenatal life in women with Graves’ disease compared to women with Hashimoto thyroiditis, which corresponds with the type of autoimmune inflammatory reaction (Th1 – mediated by estrogens in Hashimoto thyroiditis and Th2 – promoted by androgens in Graves’ disease). This may indicate a different role of prenatal sex hormones in the etiology of these diseases, both of which are autoimmune but they differ in the type of immunological response and a balance of Th1 and Th2 lymphocytes.

Digit ratio (2D:4D) is thought to be a proxy of prenatal androgen and estrogen exposure. In some populations, the associations between 2D:4D and prenatal sex steroids are greater for the right hand than for the left hand (31, 32). Similarly, right-left 2D:4D (Δ r–l 2D: 4D) is thought to be negatively correlated with high prenatal testosterone and low prenatal estrogen (31, 33). In this study, we found significant differences for right and left 2D:4D, but also right 4D appeared to be significantly shorter in women with Hashimoto thyroiditis than in Graves’ disease. This relation had greater effect size for the right than left 2D:4D. The postulated relationships between 2D:4D and prenatal sex steroids and the stability of 2D:4D during childhood and puberty has been explored in detail for more than 20 years (18–20, 22, 23). The early formation of the thyroid gland and the modulation of its function by sex steroids (27) led Manning to suggest that 2D:4D may be a correlate of thyroid function and thyroid disease (25). We emphasize that our main purpose here was to report that 2D:4D is a correlate of AITD. Insofar as our findings arose from the hypothesis that 2D:4D is a correlate of prenatal sex steroids our data on 2D:4D and AITD may be considered as further evidence of the link between 2D:4D and fetal testosterone/estrogen balance. However, there may be other possibilities to consider. Compared to controls, we found BMI was higher in women with AITD. It has been suggested that 2D:4D and its sex differences are influenced by both the length of the phalanges and by fat pads at the tip of the digits (34). Therefore, adiposity might provide a link between 2D:4D and thyroid disease. Furthermore, Wallen has hypothesized that the sex difference in 2D:4D is not present in the phalanges rather it lies solely in variation in digit fat pads and the latter are not correlates of prenatal sex steroids (35). Against Wallen’s hypothesis, sex differences in phalange 2D:4D have been reported in predominantly White fetuses and in White, Asian and Black populations (for reviews of the literature see (36, 37). The differences we have found in mean 2D:4D between controls and patients with Hashimoto and Graves’ disease are independent of BMI. In our view there is little support in our results for a link between finger fat, 2D:4D and AITD.

Focusing on the fetus, we suggest the following model. Digit ratio and its sex differences are more or less fixed between week 8 and 12 in gestation. The relative lengths of 2D and 4D are influenced by the ratio between prenatal testosterone and prenatal estrogen such that high prenatal testosterone relative to estrogen is linked to low values of 2D:4D and low testosterone to estrogen is associated with high values of 2D:4D. The first trimester sex steroids which influence 2D:4D are both fetal and maternal in origin and maternal T4 (which is modulated by maternal testosterone and estrogen) crosses the placenta from the maternal serum. Thus, fetal 2D:4D contains information concerning prenatal concentrations of testosterone, estrogen and T4.

**Figure 1:**
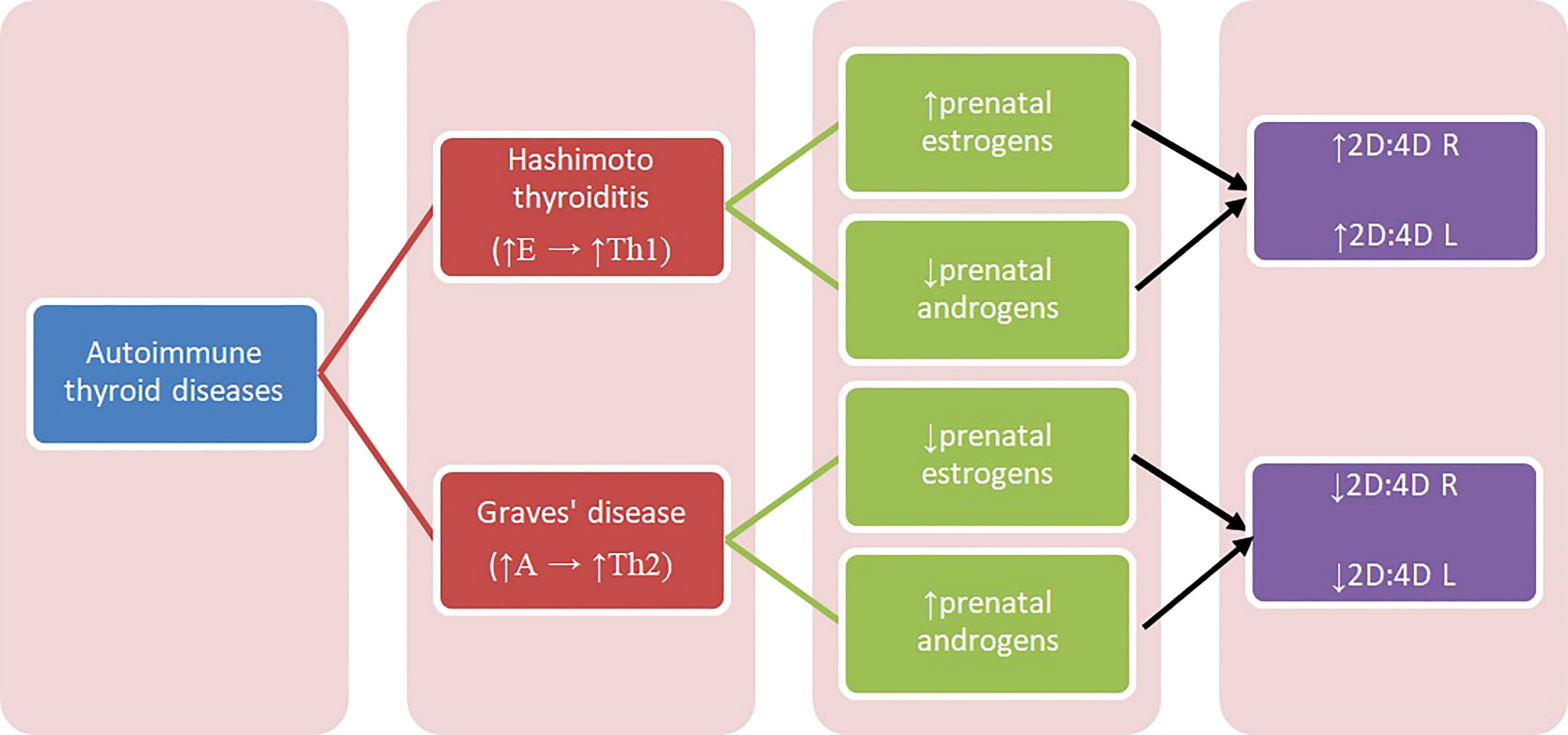
Diagram presenting relation between autoimmune thyroid diseases and 2D:4D. A – androgens, E – estrogens, Th1, T-helper 1 cells; Th2, T-helper 2 cells; 2D:4D R, right digit ratio; 2D:4D L, left digit ratio.

Concerning Hashimoto and Graves’ disease, our results may be interpreted with regard to two aspects: sex hormones influence on thyroid function and their modulation of immunologic response, also in relation to autoimmunologic mechanisms. In some conditions, estrogens can stimulate the immune system, while in others they have an inhibitory effect. Estrogen receptor alpha (ERα) and beta (ERβ) are two main types of 17β-estradiol (estrogen) nuclear receptors which play a relevant role in transcriptional regulation (32). ERα and ERβ are present in immune system cells, including CD4+ and CD8+ lymphocytes, suggesting that T cells can be amenable to regulation by estrogen (38–40). ERα-signaling can be potentially pro-inflammatory in T cells, increasing expression of transcription factor Tbx21, which is associated with Th1 (39–42). Overall, estrogens increase Th1 differentiation and decrease Th2 (17). Th1 lymphocytes stimulated by high estrogen levels can result in Th1-mediated diseases, one of which is Hashimoto thyroiditis. We hypothesize that higher prenatal estrogen exposure promotes autoimmune response development in adulthood and predisposes to Hashimoto thyroiditis (**Figure 1**).

There are no studies concerning prenatal sex steroid influence on Hashimoto thyroiditis and Graves’ disease, and the reports on prenatal estrogen’s influence on autoimmune diseases are also limited. Previous studies showed that patients with systemic lupus erythematosus (SLE) (both women and men) had lower 2D:4D than healthy controls (43). The authors concluded that high prenatal levels of testosterone and low estrogen can play a role in the etiology of this disease onset and can be its trigger (43). Furthermore, they hypothesize that increased aromatase activity can cause an increased level of estrogen, which can lead to the disease’s commencement (43). Moreover, because SLE is considered to be a disease in which Th2 cells predominate, the observed correlation can be explained by increased Th2 response promoted by increased androgens’ exposure (17). Similar mechanism may explain our findings in women with Graves’ disease. Kocjan et al. confirmed the important role of thyroid stimulating hormone receptor (TSHR) antibodies and humoral immunity in Graves’ disease pathogenesis, and showed the systemic shift of cytokines production toward the Th2 response (13) (**Figure 1**).

The second possible aspect of sex hormone’s influence on thyroid function includes postnatal estrogens influence on the thyroid gland. ERα and ERβ have been detected in thyroid gland tissue- normal and neoplastic. The imbalance between estrogen receptors α and β may have a role in thyroid cancer development (44). The potential impact of ER β as tumor-suppressive in thyroid cancer has also been described (45–47).

The association between Hashimoto thyroiditis, TPOAb and obesity was supported in systemic review and meta-analysis (48). Our research showed that women with Hashimoto thyroiditis had significantly higher BMI than the controls, however our analysis showed the observed correlations were independent of BMI.

We suggest that relationships between first trimester maternal sex steroids and T4 and children’s 2D:4D should be examined. In addition, associations between 2D:4D, T4 and TSH should be considered in healthy children and adults. We predict that 2D:4D will be positively correlated with TSH. With regard to AITD’s, the main limitation of our research is the number of the studied participants with Hashimoto thyroiditis and Graves’ disease and its restriction to only female subjects. What is more, there are differences in patients’ age: women with Graves’ disease were older than those with Hashimoto thyroiditis and control women. On the other hand, the mean age of Graves’ disease onset is higher than for Hashimoto thyroiditis and 2D:4D digit ratio is independent of age (18). Theoretically, younger women from the control group may develop Graves’ disease in the future, however this disease is rare, so it seems most unlikely that this may occur. A study including patients of both sexes as well as patients with and without thyroid cancer would be beneficial. Also, similar studies in ethnic groups other than Caucasians should be performed.

In conclusion, women with Hashimoto thyroiditis have higher 2D:4D, and women with Graves’ disease have lower 2D:4D, than women without the diseases. Higher prenatal exposure to estrogens may play a role in the development of Hashimoto thyroiditis (e.g. by being indicative of higher sensitivity of estrogen receptors). In contrast, higher prenatal exposure to androgens (or lower to estrogens) may predispose to Graves’ disease. Further studies on the influence of prenatal sex hormones on vulnerability to develop autoimmune thyroid diseases are needed.

## Data Availability Statement

The database associated with the article is available in the FigShare repository under a link: https://doi.org/10.6084/m9.figshare.16803055.

## Ethics Statement

The studies involving human participants were reviewed and approved by the Ethics Committee of Medical University of Lodz (RNN/04/19KE, 15.01.2019). The patients/participants provided their written informed consent to participate in this study.

## Author Contributions

BS: conception, drafting the work, and analysis of data, acquisition of the data. AK-T: conception, drafting the work, acquisition and interpretation of data for the work, review and editing. JM: conception, supervision, interpretation of data for the work, review and editing. BA: conception, supervision, project administration. All authors contributed to the article and approved the submitted version.

## Conflict of Interest

The authors declare that the research was conducted in the absence of any commercial or financial relationships that could be construed as a potential conflict of interest.

## Publisher’s Note

All claims expressed in this article are solely those of the authors and do not necessarily represent those of their affiliated organizations, or those of the publisher, the editors and the reviewers. Any product that may be evaluated in this article, or claim that may be made by its manufacturer, is not guaranteed or endorsed by the publisher.

## References

[1] Tomer Y Huber A. The Etiology of Autoimmune Thyroid Disease: A Story of Genes and Environment. J Autoimmun (2009) 32(3-4):231–9. doi: 10.1016/j.jaut.2009.02.007 PMC3561494 19307103

[2] Hashimoto’s Thyroiditis (2013). Available at: http://www.thyroidmanager.org/chapter/hashimotos-thyroiditis/ (Accessed May 30,2021).

[3] Jóźków P Lwow F Słowińska-Lisowska M Mędraś M. Trends in the Prevalence of Autoimmune Thyroiditis in the Leading Private Health-Care Provider in Poland. Adv Clin Exp Med (2017) 26(3):497–503. doi: 10.17219/acem/60862 28791826

[4] Pyzik A Grywalska E Matyjaszek-Matuszek B Roliński J. Immune Disorders in Hashimoto's Thyroiditis: What Do We Know So Far? J Immunol Res (2015) 2015:979167. doi: 10.1155/2015/979167 26000316 PMC4426893

[5] Caturegli P De Remigis A Rose NR. Hashimoto Thyroiditis: Clinical and Diagnostic Criteria. Autoimmun Rev (2014) 13(4-5):391–7. doi: 10.1016/j.autrev.2014.01.007 24434360

[6] Arduc A Aycicek Dogan B Bilmez S Imga Nasiroglu N Tuna MM Isik S. High Prevalence of Hashimoto's Thyroiditis in Patients With Polycystic Ovary Syndrome: Does the Imbalance Between Estradiol and Proges-Terone Play a Role? Endocr Res (2015) 40(4):204–10. doi: 10.3109/07435800.2015.1015730 25822940

[7] Berger A. Th1 and Th2 Responses: What Are They? BMJ (2000) 321(7258):424. doi: 10.1136/bmj.321.7258.424 10938051 PMC27457

[8] StatPearls. Diffuse Toxic Goiter (2017). Available at: https://www.ncbi.nlm.nih.gov/books/NBK557859/ (Accessed May 15, 2022).

[9] StatPearls. Graves Disease (2021). Available at: https://www.ncbi.nlm.nih.gov/books/NBK448195/ (Accessed May 15, 2022).

[10] Bahn RS. Graves' Ophthalmopathy. N Engl J Med (2010) 362(8):726–38. doi: 10.1056/NEJMra0905750 PMC3902010 20181974

[11] Chin YH Ng CH Lee MH Koh JWH Kiew J Yang SP. Prevalence of Thyroid Eye Disease in Graves' Disease: A Meta-Analysis and Systematic Review. Clin Endocrinol (Oxf) (2020) 93(4):363–74. doi: 10.1111/cen.14296 32691849

[12] Kahaly GJ Bartalena L Hegedüs L Leenhardt L Poppe K Pearce SH. 2018 European Thyroid Association Guideline for the Management of Graves' Hyperthyroidism. Eur Thyroid J (2018) 7(4):167–86. doi: 10.1159/000490384 PMC6140607 30283735

[13] Kocjan T Wraber B Repnik U Hojker S. Changes in Th1/Th2 Cytokine Balance in Graves' Disease. Pflugers Arch (2000) 440(5 Suppl):R94–5. doi: 10.1007/s004240000019 11005626

[14] Lahita RG. Gender and the Immune System. J Gend Specif Med (2000) 3:19–22. doi: 10.1016/s1567-5769(01)00044-3 11252923

[15] Olsen NJ Kovacs WJ. Gonadal Steroids and Immunity. Endocr Rev (1996) 17:369–84. doi: 10.1210/edrv-17-4-369 8854050

[16] Lambert KC Curran EM Judy BM Milligan GN Lubahn DB Estes DM. Oestrogen Receptor Alpha (Erα) Deficiency in Macrophages Results in Increased Stimulation of CD4+ T Cells While 17beta-Estradiol Acts Through Erα to Increase IL-4 and GATA-3 Expression in CD4+ T Cells Independent of Antigen Presentation. J Immunol (2005) 175:5716–23. doi: 10.4049/jimmunol.175.9.5716 16237062

[17] Moulton VR. Sex Hormones in Acquired Immunity and Autoimmune Disease. Front Immunol (2018) 9:2279. doi: 10.3389/fimmu.2018.02279 30337927 PMC6180207

[18] Manning JT Scutt D Wilson J Lewis-Jones DI. The Ratio of 2nd to 4th Digit Length: A Predictor of Sperm Numbers and Concentrations of Testosterone, Luteinizing Hormone and Oestrogen. Hum Reprod (1998) 13(11):3000–4. doi: 10.1093/humrep/13.11.3000 9853845

[19] Manning J Kilduff L Cook C Crewther B Fink B. Digit Ratio (2d:4d): A Biomarker for Prenatal Sex Steroids and Adult Sex Steroids in Challenge Situations. Front Endocrinol (Lausanne) (2014) 5:9. doi: 10.3389/fendo.2014.00009 24523714 PMC3906590

[20] Manning JT. Resolving the Role of Prenatal Sex Steroids in the Development of Digit Ratio. Proc Natl Acad Sci USA (2011) 108(39):16143–4. doi: 10.1073/pnas.1113312108 PMC3182713 21930921

[21] Kasielska-Trojan A Manning JT Antczak A Dutkowska A Kuczyński W Sitek A. Digit Ratio (2D:4D) in Women and Men With Lung Cancer. Sci Rep (2020) 10(1):11369. doi: 10.1038/s41598-020-68239-0 32647333 PMC7347627

[22] Zheng Z Cohn MJ. Developmental Basis of Sexually Dimorphic Digit Ratios. Proc Natl Acad Sci USA (2011) 108(39):16289–94. doi: 10.1073/pnas.1108312108 PMC3182741 21896736

[23] Swift-Gallant A Johnson BA Di Rita V Breedlove SM. Through a Glass, Darkly: Human Digit Ratios Reflect Prenatal Androgens, Imperfectly. Horm Behav (2020) 120:104686. doi: 10.1016/j.yhbeh.2020.104686 32014464

[24] McCormick CM Carré JM. Facing Off With the Phalangeal Phenomenon and Editorial Policies: A Commentary on Swift-Gallant, Johnson, Di Rita and Breedlove (2020). Horm Behav (2020) 120:104710. doi: 10.1016/j.yhbeh.2020.104710 32057822

[25] Manning J. The Finger Ratio. London: Faber and Faber (2009) 5–10, 62-63.

[26] Patel J Landers K Li H Mortimer RH Richard K. Thyroid Hormones and Fetal Neurological Development. J Endocrinol (2011) 209(1):1–8. doi: 10.1530/JOE-10-0444 21212091

[27] Tahboud R Arafah BM. Sex Steroids and the Thyroid. Best Pract Res Clin Endocrinol Metab (2009) 23(6):769–80. doi: 10.1016/j.beem.2009.06.005 19942152

[28] Tabachnik M Sheiner E Wainstock T. The Association Between Second to Fourth Digit Ratio, Reproductive and General Health Among Women: Findings From an Israeli Pregnancy Cohort. Sci Rep (2020) 10(1):6341. doi: 10.1038/s41598-020-62599-3 32286380 PMC7156723

[29] Derwahl M Nicula D. Estrogen and its Role in Thyroid Cancer. Endocr Relat Cancer (2014) 21(5):T273–83. doi: 10.1530/ERC-14-0053 25052473

[30] Erbach GT Bahr JM. Enhancement of *In Vivo* Humoral Immunity by Estrogen: Permissive Effect of a Thymic Factor. Endocrinology (1991) 128(3):1352–8. doi: 10.1210/endo-128-3-1352 1999157

[31] Manning JT Fink B Mason L Kasielska-Trojan A Trivers R. The Effects of Sex, Nation, Ethnicity, Age and Self-Reported Pubertal Development on Participant-Measured Right-Left 2D:4D (Dr-L) in the BBC Internet Study. J Biosoc Sci (2022) 1–13. doi: 10.1017/S0021932022000049 35088686

[32] Xu Y Zheng Y. The Digit Ratio (2D:4D) in China: A Meta-Analysis. Am J Hum Biol (2015) 27(3):304–9. doi: 10.1002/ajhb.22639 25284473

[33] Manning JT Fink B. “Digit Ratio and Personality and Individual Differences”. In: Zeigler-Hill V Shackelford TK, editors. The SAGE Handbook of Personality and Individual Differences. Los Angeles: SAGE Publications Ltd (2018). 40–50.

[34] Manning JT Fink B Neave N Caswell N. Photocopies Yield Lower Digit Ratios (2D:4D) Than Direct Finger Measurements. Arch Sex Behav (2005) 34(3):329–33. doi: 10.1007/s10508-005-3121-y 15971015

[35] Wallen K. Does Finger Fat Produce Sex Differences in Second to Fourth Digit Ratios? Endocrinology (2009) 150(11):4819–22. doi: 10.1210/en.2009-0986 19846613

[36] Xi H Li M Fan Y Zhao L. A Comparison of Measurement Methods and Sexual Dimorphism for Digit Ratio (2D:4D) in Han Ethnicity. Arch Sex Behav (2014) 43(2):329–33. doi: 10.1007/s10508-013-0179-9 PMC3890058 24013635

[37] Trivers R Jacobson A Manning JT. Radiographic Digit Ratios (2D:4D) of Afro-Caribbean Children: Comparisons With Published Data From White Children. Early Hum Dev (2020) 146:105072. doi: 10.1016/j.earlhumdev.2020.105072 32485482

[38] Goodman WA Bedoyan SM Havran HL Richardson B Cameron MJ Pizarro TT. Impaired Estrogen Signaling Underlies Regulatory T Cell Loss-of-Function in the Chronically Inflamed Intestine. Proc Natl Acad Sci USA (2020) 117(29):17166–76. doi: 10.1073/pnas.2002266117 PMC7382259 32632016

[39] Khan D Ansar Ahmed S. The Immune System Is a Natural Target for Estrogen Action: Opposing Effects of Estrogen in Two Prototypical Autoimmune Diseases. Front Immunol (2016) 6:635. doi: 10.3389/fimmu.2015.00635 26779182 PMC4701921

[40] Kovats S. Estrogen Receptors Regulate Innate Immune Cells and Signaling Pathways. Cell Immunol (2015) 294(2):63–9. doi: 10.1016/j.cellimm.2015.01.018 PMC4380804 25682174

[41] Maret A Coudert JD Garidou L Foucras G Gourdy P Krust A. Estradiol Enhances Primary Antigen-Specific CD4 T Cell Responses and Th1 Development *In Vivo.* Essential Role of Estrogen Receptor Alpha Expression in Hematopoietic Cells. Eur J Immunol (2003) 33(2):512–21. doi: 10.1002/immu.200310027 12645950

[42] Karpuzoglu E Phillips RA Gogal RM Jr Ansar Ahmed S. IFN-Gamma-Inducing Transcription Factor, T-Bet Is Upregulated by Estrogen in Murine Splenocytes: Role of IL-27 But Not IL-12. Mol Immunol (2007) 44(7):1808–14. doi: 10.1016/j.molimm.2006.08.005 PMC3097111 17046061

[43] Doe K Nozawa K Hirai T Tsushima H Hayashi E Hiruma K. Second-To-Fourth Digit Ratio in Systemic Lupus Erythematosus. J Rheumatol (2015) 42(5):826–8. doi: 10.3899/jrheum.140974 25729029

[44] Božović A Mandušić V Todorović L Krajnović M. Estrogen Receptor Beta: The Promising Biomarker and Potential Target in Metastases. Int J Mol Sci (2021) 22(4):1656. doi: 10.3390/ijms22041656 33562134 PMC7914503

[45] Dong W Zhang H Li J Guan H He L Wang Z. Estrogen Induces Metastatic Potential of Papillary Thyroid Cancer Cells Through Estrogen Receptor Alpha and Beta. Int J Endocrinol (2013) 2013:941568. doi: 10.1155/2013/941568 24222765 PMC3810507

[46] Zeng Q Chen GG Vlantis AC van Hasselt CA. Oestrogen Mediates the Growth of Human Thyroid Carcinoma Cells *via* an Oestrogen Receptor—ERK Pathway. Cell Prolif (2007) 40:921–35. doi: 10.1111/j.1365-2184.2007.00471.x PMC6495898 18021179

[47] Cho MA Lee MK Nam K-H Chung WY Park CS Lee JH. Expression and Role of Estrogen Receptor α and β in Medullary Thyroid Carcinoma: Different Roles in Cancer Growth and Apoptosis. J Endocrinol (2007) 195:255–63. doi: 10.1677/JOE-06-0193 17951536

[48] Song RH Wang B Yao QM Li Q Jia X Zhang JA. The Impact of Obesity on Thyroid Autoimmunity and Dysfunction: A Systematic Review and Meta-Analysis. Front Immunol (2019) 10:2349. doi: 10.3389/fimmu.2019.02349 31681268 PMC6797838

